# Chromatin Regulation of HSV Gene Transcription

**DOI:** 10.3390/v17111409

**Published:** 2025-10-23

**Authors:** Yuxuan Zheng, Juncheng Zhang, Dongli Pan

**Affiliations:** 1 State Key Laboratory for Diagnosis and Treatment of Infectious Diseases, National Clinical Research Center for Infectious Diseases, Collaborative Innovation Center for Diagnosis and Treatment of Infectious Disease, The First Affiliated Hospital, Zhejiang University School of Medicine, Hangzhou 310003, China3200101260@zju.edu.cn (J.Z.); 2 Department of Microbiology, Zhejiang University School of Medicine, Hangzhou 310058, China

**Keywords:** HSV-1, epigenetics, chromatin regulation, histone modification

## Abstract

Herpes simplex virus (HSV) has a complicated life cycle including stages of primary lytic infection, latent infection, and reactivation. Although the HSV genomic DNA within the viral capsid is devoid of histones, it rapidly associates with histones upon entering the nucleus to form viral chromatin. This chromatin is not integrated into the host chromosome and displays features distinct from the cellular chromatin. The composition, structure, and post-translational modifications of the HSV chromatin change over the course of infection due to the actions of numerous viral and host molecules. In turn, the chromatin states influence the transcription profiles of viral genes at all stages of the viral life cycle and may dictate the outcomes of the lytic-latent balance. These mechanisms may be exploited to develop new antiviral therapeutics. This review summarizes current knowledge about the formation, regulation, and functions of the HSV chromatin and discusses the questions remaining to be answered.

## 1. Introduction

Herpesviruses are large, enveloped double-stranded DNA viruses that predominantly infect vertebrate hosts, with hallmark capacities to establish lifelong latent infection and reactivate from it. Herpes simplex virus (HSV), including HSV-1 and HSV-2, are members of the alphaherpesvirus subfamily. HSV-1 primarily infects the orolabial region, causing common diseases such as cold sores and keratitis as well as rare but deadly herpes simplex encephalitis, while HSV-2 predominantly infects the genital mucosa, leading to genital herpes. Both HSV-1 and HSV-2 are also pathogens of neonatal herpes. Globally, the seroprevalences of HSV-1 and HSV-2 are estimated to be ~70% and 10%, respectively [1]. HSV undergoes productive (lytic) infection in cells of peripheral mucosal tissues and then enter neurons of the peripheral nervous system to establish latent infection. When stimulated by neural damage, UV irradiation, hyperthermia, stress hormones, inflammatory cytokines, or neurotrophic factor deprivation [2], the virus can reactivate from latency, resulting in resumed virus production in the neurons followed by virus transportation back to the original sites of infection, where recurrent diseases may manifest. Despite the availability of anti-HSV drugs, HSV disease is currently incurable largely due to the latency–reactivation cycle.

**Figure 1:**
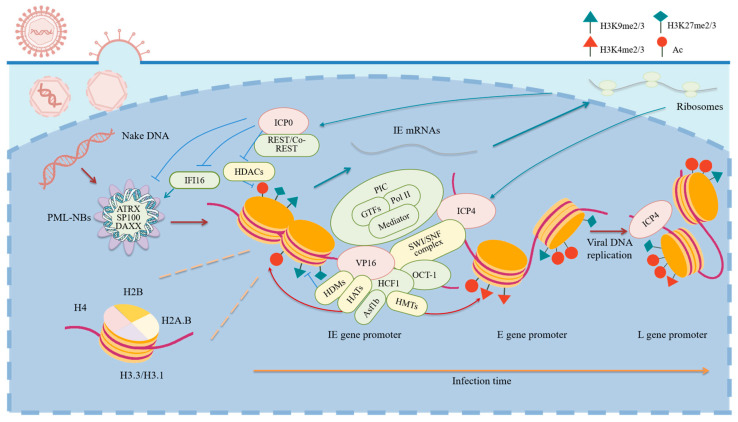
Chromatin regulation of HSV-1 gene transcription during lytic infection. Upon nuclear entry, incoming viral DNA associates with host histones and repressive heterochromatin modifications. This transient silencing is counteracted by viral proteins such as VP16, which complexes with OCT-1 and HCF-1 to recruit the pre-initiation complex (PIC), including general transcription factors (GTFs), Mediator, and RNA polymerase II (Pol II) to IE gene promoters. The VP16 induced complex also recruits chromatin remodeling complexes, such as the SWI/SNF complex, histone methyltransferases (HMTs), histone acetyltransferases (HATs), and histone demethylases (HDMs). ICP0 disrupts PML-NBs and REST/Co-REST, and induces the degradation of IFI16 to facilitate transcriptional activation. ICP4 also recruits PIC and the SWI/SNF complex to E/L gene promoters, resulting in decreased histone occupancies and increased chromatin accessibility. These coordinated events ensure the ordered expression of HSV-1 genes and efficient virion production. Created with BioGDP.com (accessed on 17 October 2025) [92].

HSV exhibits distinct gene expression programs at different stages of infection. During lytic infection, entry of the viral genome into the nucleus is followed by gene transcription in a cascade manner: immediate–early (IE), early (E), and late (L) genes are sequentially activated (Figure 1). At the beginning, IE genes are activated by viral tegument protein virion protein 16 (VP16) complexed with cellular proteins octamer-binding transcription factor 1 (OCT-1) and host cell factor 1 (HCF-1) [3,4,5]. The expressed IE proteins then antagonize host restrictive mechanisms and activate the expression of E and L genes. E genes encode proteins required for viral DNA synthesis. L genes mostly encode viral structural proteins and are expressed efficiently only after viral DNA replication has started. Such a lytic cycle can occur in a broad spectrum of cell types. However, in HSV-1 infected neuronal tissues in vivo and neuronal culture treated with an inhibitor of viral DNA synthesis, latency is established [6,7]. HSV latency is usually thought to be neuron-specific at least in vivo although non-neuronal cells can be used to generate quiescent infection models mimicking the gene silencing process [8]. The viral gene expression profile during latency is generally characterized by global silencing of all viral genes except the latency-associated transcript (LAT) region. However, the real situation is likely to be more complicated due to the heterogeneity of infected neurons. Lytic transcripts and even proteins could be detected in a small subset of latently infected mouse ganglionic neurons despite no evidence of infectious virus particles produced [9] and LATs were not detected in all latently infected human neurons [10]. Regardless, the latent gene expression program can be perturbed by certain stimuli to initiate reactivation, which is generally characterized as a two-step process: global de-repression of the viral genome independent of VP16, followed by VP16-dependent ordered gene expression leading to production of infectious viral particles [11].

A major factor that determines the viral gene expression profile is the chromatin state of the HSV genome. In eukaryotes, DNA wraps around histone octamers to form nucleosomes, which are fundamental units of chromatin. During HSV infection, the viral genome can also bind host histones to assemble into functionally relevant chromatin. The compositions, structures, and modifications of HSV chromatin are regulated by various viral and host factors, and therefore constantly change over the course of infection. The chromatin states in turn profoundly influence the transcription profile of viral genes. This article summarizes the current knowledge about the characteristics, regulators, and functions of HSV chromatin and their implications in viral gene transcription. We note that there is substantial evidence that HSV infection reshapes cellular chromatin that may influence viral infection indirectly [12]. However, this article focuses on the viral chromatin. We also note that almost all current literature has focused on HSV-1, but given the similarities between HSV-1 and HSV-2, some conclusions may be applicable to HSV-2.

## 2. Dynamic Association of Histones with the HSV Genome

### 2.1. Histone Association During Lytic Infection

After docking at the nuclear pore complex, the viral capsid releases the viral genome into the nucleus. High resolution imaging data demonstrated that the HSV-1 genome is delivered into the cellular nucleus in a pre-existing compact state [13]. Once inside the nucleus, the viral genome rapidly associates with histones to form chromatin [14,15,16]. In human neuroblastoma SY5Y cells, the association of histones with the viral genome can be detected as early as 1 h post infection (hpi), and peaks at around 3 hpi [14]. The formed viral chromatin is structurally distinct from host chromatin. Imaging analysis revealed a marked underrepresentation of histones on viral DNA relative to cellular DNA at 2 hpi in African green monkey Vero cells [17]. Assay for transposase-accessible chromatin with high-throughput sequencing (ATAC-seq) data in human fetal lung fibroblast MRC5 cells further demonstrated that the HSV-1 genome is significantly more accessible than host genome at 2 hpi, allowing RNA polymerase II and other DNA-binding proteins to readily bind [18]. Once formed, the viral chromatin undergoes constant transformation. 5-Ethynyl-2′-deoxycytidine (EdC)-labeling experiments in retinal pigment epithelial (RPE-1) cells indicated that from 0.5 to 2 hpi, EdC signals, representing HSV-1 genomes, became more diffuse, implying chromatin relaxation, which might prime the genome for subsequent transcription [19]. From 3 to 6 hpi, histone association with the viral genome decreases markedly, correlating with increased chromatin accessibility [14,20,21,22]. Notably, by 6 hpi, viral DNA replication has started. DNA replication may dilute histones associated with each viral genome, thereby further increasing chromatin accessibility. In turn, low histone association should facilitate DNA replication as well as transcription. Indeed, in Vero cells treated with the viral replication inhibitor phosphonoacetic acid (PAA), the viral DNA forms nucleosome-like complexes at 7 hpi, and overall viral chromatin accessibility is reduced relative to cells without treatment [22] suggesting that viral DNA replication corresponds with reduced chromatin accessibility. Accordingly, micrococcal nuclease digestion assays showed that most HSV-1 DNA becomes highly accessible in Vero cells by 8 hpi, even with some regions appearing as naked DNA [22,23]. Furthermore, isolation of proteins on nascent DNA (iPOND), which is a proteomic method of identifying DNA-associating proteins, failed to detect histones associated with replicating viral DNA after 6 hpi in MRC5 cells suggesting that the HSV-1 genome is replicated mainly in a histone-free state [17]. These findings suggest that the HSV-1 genome associates with histones to limited extents during lytic infection with particularly low association at late times post-infection. One caveat of this conclusion is that most of these experiments were performed in non-neuronal cells, so whether neuronal lytic infection follows the same pattern needs further clarification.

Each canonical nucleosome consists of two molecules each of H2A, H2B, H3, and H4 [24]. All these histones are mobilized during HSV-1 infection, hinting at their active participation of the viral chromatin assembly [25,26]. Each of these histones has different variants that play different roles in chromatin assembly. Most studied are H3 variants, among which H3.3 has been demonstrated to be assembled into HSV-1 chromatin at promyelocytic leukemia nuclear bodies (PML-NBs) at the initial stage of infection without requiring viral DNA replication [27], whereas H3.1 is incorporated later dependent on viral DNA replication [25,28]. H2A variants and H2B are assembled into HSV-1 chromatin too but the H2A.B variant seems more enriched than canonical H2A [29]. Notably, recent studies using high-resolution imaging demonstrated a substantial degree of population heterogeneity in the localization of histones in that H3.3 and H4 exhibited much higher frequencies of colocalization with viral DNA than H2A, H2B and H3.1 at 1.5 hpi in human foreskin fibroblast cells, leading to a model of sequential histone loading, where histone H3/H4 heterodimers are loaded onto viral DNA as a tetrasome prior to histone H2A/H2B incorporation [13].

### 2.2. Histone Association During Latency

In contrast to the situation during lytic infection, in vivo studies analyzing mouse brainstems and trigeminal ganglia showed that histones gradually accumulate on the viral genome as latency is being established in neurons [30,31], ultimately leading to formation of the latent viral chromatin characterized as viral episomes associated with nucleosomes [32,33,34]. During latency, the HSV-1 genome is associated with PML-NBs, forming viral DNA-containing PML-NBs, within which H3.3, but not H3.1, is the dominant H3 variant assembled into viral chromatin [8].

## 3. Dynamic Changes to Histone Modifications on HSV Chromatin

Histone modifications provide binding platforms for transcriptional regulators, including chromatin remodeling factors, histone chaperones, DNA/histone-modifying enzymes, and general transcription factors. These modifications influence chromatin structures by affecting either the interactions between histones of neighboring nucleosomes or the interactions between histones and DNA [35,36]. Histones bearing both repressive and activating modifications are associated with the HSV-1 genome. Histone methylation recruits “reader” proteins to either promote or repress transcription, depending on the histone residues modified. Methylations at lysine 9 or 27 of histone H3 (H3K9 or H3K27) are hallmarks of repressive heterochromatin [37], whereas methylation at lysine 4 (H3K4) is a hallmark of transcriptionally active chromatin [38,39]. Histone acetylation can generally disrupt DNA-histone interactions as well as interactions between neighboring nucleosomes [40,41], thereby changing chromatin structures and accessibility [42], and is generally associated with open chromatin and active transcription [43,44].

### 3.1. Histone Modifications During Lytic Infection

Histone modifications associated with the HSV-1 genome can be detected by 1 hpi [45], but their levels change over the course of infection (Figure 1). After being normalized to total H3, the levels of heterochromatin modifications such as H3K9me3 and H3K27me3 at viral DNA are stable from 1 to 4 hpi before their decline afterward in human foreskin fibroblast (HFF) cells [46]. By contrast, at least in monocyte THP-1 cells, H3K27ac showed an increase in enrichment at viral DNA from 4 to 8 hpi. Both the decreases in H3K9me3 and H3K27me3 and the increase in H3K27ac associated with viral DNA have the anticipated effects of increasing chromatin accessibility, and therefore are likely conducive to viral gene activation.

Histone ubiquitination is also regulated during HSV-1 infection. The viral E3 ubiquitin ligase ICP0 induces proteosomal degradation of host E3 ubiquitin ligases RNF8 and RNF168, resulting in a global reduction in H2A ubiquitination [47]. This process interferes with the recruitment of DNA damage response factors and promotes viral replication. Since ubiquitinated H2A is linked to transcriptional repression, the loss of H2A ubiquitination might also contribute to chromatin-mediated derepression of the viral genome.

### 3.2. Histone Modifications During Latency and Reactivation

**Figure 2:**
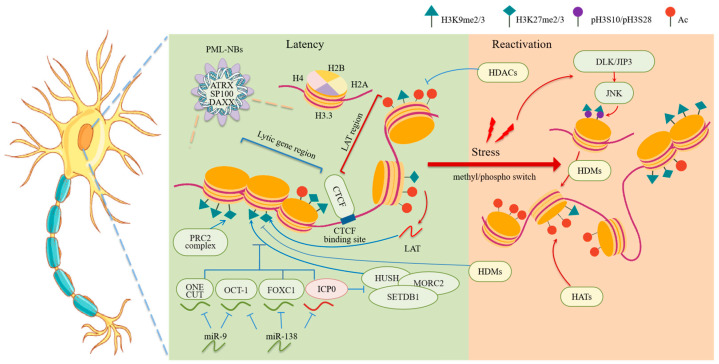
Chromatin regulation of HSV-1 gene transcription during latency and reactivation. HSV-1 establishes latent infection in the neuronal cell bodies. During latency, the HSV-1 genome is associated with PML-NBs. The latent viral chromatin is enriched with repressive histone marks such as H3K9me2/3 and H3K27me3. Activating histone modifications (H3K9ac, H3K14ac and H3K4me2/me3) are more enriched on the LAT region than lytic regions. PML-NB components such as ATRX and DAXX, as well as the MORC2 and PRC2 complexes might be involved in the formation or maintenance of the repress viral chromatin during latency. CTCF may function as an insulator that separate regions with different chromatin states. Some neuron-specific host miRNAs target activating viral and host factors to promote repressive chromatin. Stress-induced JNK-mediated pathways triggers reactivation by causing a histone methyl/phospho switch in viral chromatin, followed by further chromatin modulation mediated by HDMs and HATs. Created with BioGDP.com (accessed on 17 October 2025) [92].

During latent infection in mouse ganglia in vivo, activating histone acetylation modifications (H3K9ac, H3K14ac and H3K4me2/me3) are more enriched on the LAT promoter and enhancer than lytic gene promoters [48,49], thereby dividing the genome into regions that are either transcriptionally permissive or repressive (Figure 2). Meanwhile, lytic gene promoters are enriched with marks of both constitutive and facultative heterochromatin [50]. Constitutive heterochromatin is characterized by enrichment of H3K9me2 and H3K9me3, which are stably distributed across the latent HSV-1 genome and function to continuously suppress gene transcription [31,50,51]. Facultative heterochromatin is usually associated with reversible gene silencing and marked by the presence of H3K27me3 and the histone variant macroH2A, both of which are incorporated into HSV-1 chromatin [30,51]. Interestingly, H3K27me3 enrichment at lytic gene promoters was low at 7 dpi but increased dramatically by 14 dpi [30] implying that it may function mainly during latency maintenance rather than the initial process of latency establishment.

Reactivation from latency not only is accompanied by but also requires changes in HSV-1 chromatin modifications. Studies in neuronal culture and in vivo models reveals a reactivation process with two phases (Figure 2). During the first phase, the stress-activated JNK signaling pathway mediates a histone methyl/phospho switch—phosphorylation of histone H3 at serine 10 (H3S10p) that antagonizes the repressive H3K9me3 mark—which triggers global de-repression of the viral genome [52,53]. The second phase of reactivation requires histone demethylase activities [54,55,56]. Furthermore, during reactivation in vivo, deacetylation of the LAT region occurs in parallel with increased histone acetylation at ICP0 and ICP4 promoters [57].

## 4. Regulation of HSV Chromatin by Viral Proteins and LATs

Because a major function of the cellular chromatin machinery in the face of foreign DNA invasion is repression of gene expression from the DNA, HSV largely relies on its own proteins to remodel the viral chromatin to activate gene transcription (Figure 1). Multiple viral proteins play such a role. These viral proteins often act through their host cofactors or targets.

VP16 is a viral tegument protein essential for HSV-1 replication and reactivation from latency [58,59,60]. As mentioned above, the VP16-HCF-1-OCT-1 complex binds to the HSV IE gene promoters to stimulate their transcription [61]. The mechanisms might be related to VP16 interactions with the pre-initiation complex, including RNA polymerase II, general transcription factors and mediators as well as multiple chromatin modifying enzymes and remodeling proteins such as CBP/p300, histone demethylases and ATP-dependent chromatin remodeling proteins of the SWI/SNF complex [15,62], although the relative importance of each of these interactions for VP16 functions needs to be further clarified.

ICP0 is a viral IE protein required for efficient HSV replication and reactivation [63]. Its functions are largely attributed to its role as an E3 ubiquitin ligase that can induce proteosomal degradation of repressive host factors [64,65]. With regard to chromatin modulation, ICP0 has been shown to not only induce the removal of histone H3 and its heterochromatin modifications from the viral genome, but also promote H3 acetylation during lytic infection [20,46,66]. The mechanisms that mediate these effects have not been fully elucidated. They may be related to the ability of ICP0 to disperse PML-NBs [67] and induce the degradation of restriction factors within the PML-NBs such as PML and Sp100 [68,69,70]. Moreover, ICP0 can also dissociate histone deacetylases (HDACs) from the REST/Co-REST repressor complex [71,72], and induce the degradation of MORC family CW-type zinc finger 2 (MORC2) to alleviate HSV-1 repression mediated by H3K9me3 [73]. Additionally, ICP0 induces the degradation of interferon gamma inducible protein 16 (IFI16), which helps maintain heterochromatin on the viral genome [74,75]. Interestingly, in latently infected mouse ganglia, ICP0 increases the association of H3 and its heterochromatin modifications with the viral genome raising the possibility that ICP0 plays dual roles at different stages of infection [76].

Another IE protein, ICP4, is a viral transcription factor essential for the expression of E and L genes [77,78,79]. ICP4 extensively interacts with the viral genome at multiple stages of infection [80] and enhances histone dynamics especially within viral replication compartments [81]. ICP4 binds as a homodimer to the viral genome located at PML-NBs [82], and recruits general transcription factors, RNA polymerase II and mediator to the E and L gene promoters [18,80,83,84]. As viral genome replication proceeds, the amount of ICP4 bound decreases [18]. ICP4 may also associate with ATP-dependent chromatin remodeling factors in the SWI/SNF, Nurd, and Ino80 chromatin remodeling complexes according to proteomic data and potentially regulate viral chromatin through these complexes [85].

ICP8 is an E protein required for viral DNA replication and L gene expression [86]. It colocalizes with replicating viral genomes and functions as a single-stranded DNA binding protein that can unwind viral DNA [19,87]. Among its interacting proteins identified by immunoprecipitation and mass spectrometry, there were multiple chromatin remodeling proteins such as BRM, BRG1 and HDAC2 [88] suggesting that ICP8 may also regulate viral chromatin besides its role in DNA replication.

While the above viral proteins modulate chromatin in favor of gene activation, the viral long non-coding RNAs, LATs, appear to have opposite effects because LAT-deletion mutant viruses show reduced association of heterochromatin marks such as H3K9me2, H3K9me3, and H3K27me3, but increased association of an euchromatin mark H3K4me3 with the viral genome [31,51]. These effects correlate with repressive effects of LATs on viral lytic gene expression [89,90,91].

## 5. Regulation of HSV Chromatin by Host Proteins and MicroRNAs (miRNAs)

HSV chromatin assembles and functions mainly based on the existing host chromatin machinery, including not only the various histone variants, but also histone chaperons as well as “writer”, “reader” and “eraser” proteins of each histone modification. Therefore, host factors are crucial for the assembly, modulation, and function of viral chromatin. Yet we have only started to understand the roles of a few host factors.

The HSV-1 genome colocalizes with PML-NBs at the initial stage of infection and during quiescent/latent infection [93]. PML-NBs contain multiple factors that can promote the deposition of histones bearing repressive modifications onto viral chromatin. Among these factors are death domain associated protein (DAXX), alpha-thalassemia/mental retardation X-linked (ATRX) and the human silencing hub (HUSH) complex [8]. While ATRX recognizes H3K9me3 specifically [94], the histone chaperone DAXX deposits new H3.3-H4 complex onto chromatin while further promoting H3K9me3 catalysis on the new histone dimer by recruiting histone methyltransferases [95]. Thus, DAXX and ATRX form an ATP-dependent SNF2-like chromatin remodeling complex [96] that propagate heterochromatin on the viral genome. During HSV-1 lytic infection, DAXX-mediated H3.3 deposition on the HSV-1 genome suppresses genome decompaction and IE gene transcription [13]. Although ATRX is dispensable for de novo deposition of H3 onto HSV genomes, it restricts infection through maintenance of viral heterochromatin [97]. The HUSH complex and its effectors SETDB1 and MORC2 are also crucial for maintaining H3K9me3 on the PML-NBs-associated HSV-1 genome [73]. Besides the PML-NB components, IFI16 can also restrict HSV-1 gene expression and replication in interferon-treated cells, which is achieved by IFI16 promoting the maintenance of viral heterochromatin [75]. Correlating with these chromatin regulatory functions, all these proteins have been shown to restrict HSV-1 replication at least under conditions where ICP0 is deleted or interferons are supplemented. Although the effects of most of these host factors on latency and reactivation have yet to be tested, at least knockdown of PML in the presence of type I interferon has been shown to increase reactivation in mouse neuronal culture [98] supporting its importance for HSV latency.

HSV has the property of establishing latency specifically in neurons. Accordingly, neuron-specific host miRNAs have recently emerged as factors contributing to viral latency and heterochromatin formation or maintenance (Figure 2). Our research found that miR-138 and miR-9 that are highly expressed specifically in neuronal cells could increase the association of histone H3 and its heterochromatin modifications with the HSV-1 genome [66,99]. Mechanistically, miR-138 targets host OCT-1 and FOXC1 as well as viral ICP0, while miR-9 targets OCT-1 and ONECUT family members [100,101]. Besides the chromatin regulatory roles of OCT1 (as a cofactor of VP16 and HCF-1) and ICP0 mentioned above, ONECUT family members and FOXC1 can also reduce the occupancies of H3 and its heterochromatin modifications on the viral genome, correlating with their ability to strongly stimulate lytic replication in neuronal cells. The mechanisms by which ONECUT family proteins and FOXC1 regulate HSV-1 chromatin are under investigation.

Host factors also work with viral proteins to promote activating chromatin modifications as demonstrated by the role of the VP16 cofactor HCF-1. HCF-1 recruits H3K9 demethylases (LSD1 and JMJD2) and a H3K4 methyltransferase (SET1) to the viral IE genes [56,102,103,104]. Moreover, HCF-1 interacts simultaneously with both viral DNA replication proteins and the histone H3/H4 chaperone Asf1b suggesting that it can also modulate viral chromatin during DNA replication [105]. Consistent with its role as a viral chromatin derepressor, HCF-1 knockout in a mouse model resulted in increased association of H3K9me3 with the viral genome after stimulation of reactivation from latency correlating with reduced rates of reactivation [106].

The HSV chromatin is not homogenous along the viral genome. At least the LAT region has been shown to associate with different levels of histone modifications and transcription activities relative to certain lytic regions tested [48,49]. One factor that is hypothesized to contribute to the region-specific chromatin states is CTCF (Figure 2). It is a ubiquitously expressed and highly conserved zinc-finger protein [107]. Upon sequence-specific DNA binding, CTCF functions as an insulator that separates adjacent domains of active and inactive chromatin by limiting interactions between enhancers and promoters and preventing the spread of heterochromatin [108]. Computational analyses identified seven CTCF binding sites located within the repeat regions encompassing the ICP0, ICP4, and LAT loci [109]. CTCF associates with these sites during latency but the association decreases after the application of reactivation stimuli [110]. Depletion of CTCF resulted in increased reactivation in a rabbit model [111]. Deletion of the CTRL2 site of CTCF binding resulted in not only altered occupancy patterns of H3K27me3 in latently infected mouse ganglia as revealed by chromatin immunoprecipitation (ChIP)-seq analysis [112], but also altered chromatin contacts within the HSV-1 genome in quiescently infected human neuronal cells as identified by 4C-seq analysis [113]. Functionally, CTRL2 deletion resulted in either decreased establishment of latency or decreased reactivation from latency in mouse models according to different reports [112,114].

## 6. Effects of Viral Chromatin on HSV Gene Transcription

Multiple lines of evidence indicate that viral chromatin plays a critical role in HSV-1 gene transcription in multiple stages of the viral life cycle. The above sections already mention many correlations between the viral chromatin states and transcription states. For instance, gene activation during lytic infection correlates with decreased association of histones and its heterochromatin modifications but increased association of histone acetylation with the viral genome, while gene silencing during the establishment of latency coincides with the accumulation of histone H3 and its heterochromatin modifications on the viral genome. Moreover, as mentioned above, multiple viral and host factors that can regulate viral replication and/or the lytic-latent switch have corresponding effects on viral chromatin implying that the effects on the viral outcomes might be mediated through the chromatin regulatory functions.

Besides the correlations, more direct evidence came from studies showing that inhibitors of proteins responsible for adding or removing histone modifications impact the outcomes of viral infection. For example, a general inhibitor of histone deacetylases, trichostatin A (TSA), stimulates HSV-1 reactivation from latency in both mouse neuronal culture and explanted mouse ganglia [115,116]. P300 (EP300) is an acetyltransferase mediating acetylation mainly at H3K18 and H3K27 sites [117]. A P300 inhibitor, C646, inhibited HSV-1 replication and gene expression in human monocyte THP-1 cells [45]. These results consistently indicate that histone acetylation promotes HSV-1 gene transcription during both lytic infection and reactivation, likely owing to its ability to loosen the structures of chromatin so that RNA polymerase II and activating proteins can readily bind. LSD1 (KDM1A) is a histone demethylase with activities at both H3K4 and H3K9 sites [118,119]. LSD1 inhibition by trans-2-phenylcyclopropanamine (TCP) resulted in suppressed HSV-1 replication and reactivation from latency in both mouse and rabbit models [55,56]. Another LSD1 inhibitor, OG-L002, had similar effects in a mouse model [120]. JMJD2 is a histone demethylase with activities at H3K9 and H3K36 sites [121]. The JMJD2 inhibitor ML324 suppressed HSV-1 replication and IE gene expression in cell culture as well as reactivation from latency in explanted mouse trigeminal ganglia [122]. Since LSD1 and JMJD2 commonly target H3K9me3, their similar effects imply that H3K9me3 represses viral replication and reactivation likely by promoting heterochromatin on the viral genome. UTX (KDM6A) and JMJD3 (KDM6B) are H3K27-specific histone demethylases. Their inhibitor GSK-J4 repressed HSV-1 gene expression in fibroblasts and reactivation from latency in sensory neurons in culture [123,124] suggesting that H3K27me3 represses viral gene expression during replication and reactivation possibly by promoting heterochromatin too. However, inhibitors of the histone H3K27me3 methyltransferases EZH1 and EZH2 (GSK126, GSK343, and UNC1999) could also repress HSV-1 replication and IE gene expression in fibroblasts, which was explained by their effects of activating host immunity rather than direct effects on viral chromatin [125]. Suppressive effects of these inhibitors on viral replication during acute infection and reactivation in a mouse model were also observed [125]. However, another study observed no effect of UNC1999 on viral gene expression in fibroblasts [124]. Therefore, the role of H3K27me3 appears less clear or context dependent. Regardless, more work is needed to clarify the effects of viral chromatin on HSV gene transcription.

## 7. Conclusions and Future Perspectives

After decades of research, the dynamic changes in viral chromatin and its upstream and downstream regulatory mechanisms have become increasingly elucidated (Figure 1 and Figure 2). It is now understood that chromatin-mediated transcriptional regulation operates throughout all stages of the HSV-1 life cycle. Upon nuclear entry, the viral genome is entrapped in PML-NBs, where components such as DAXX facilitate deposition of histones onto the viral genome. The resulting viral chromatin is initially enriched with host restriction factors that promote heterochromatin formation and gene silencing. Viral regulatory proteins counteract this repression through distinct mechanisms: VP16 forms a complex with host OCT-1 and HCF-1 to recruit activating histone remodeling and modifying proteins to IE promoters to stimulate IE gene expression; ICP0 induces degradation of components of PML-NBs and chromatin-modifying complexes, among other mechanisms, to mitigate the host restrictive mechanisms; ICP4 recruits pre-initiation complex to promote transcription of E and L genes. During latent infection in neurons, low expression of the viral activators combined with high expression of viral LATs as well as host neuron-specific and immune factors favors the establishment and maintenance of heterochromatin along the viral genome, thereby facilitating silencing of viral genes although the LAT region is transcriptionally active due to reasons not completely understood. Stress-induced activation of a histone methyl/phosphor switch followed by actions from histone demethylases reverses this repression, enabling transcriptional reactivation. These interconnected epigenetic processes orchestrate the dynamic equilibrium between HSV-1 latency and reactivation.

Notably, the advancement of this field is at least in part attributable to the rapidly developing techniques in chromatin research. While traditional methods such as ChIP-qPCR can quantify enrichment of histones and their modifications at certain loci, they offer little information about their genomic distribution. This gap is filled by high-throughput sequencing in methods such as ChIP-seq (e.g., [112]). While ChIP-seq is often limited in sensitivity and requires large numbers of cells, CUT&Tag incorporates Tn5-transposase-based tagging to increase sensitivity [126,127]. Tn5-transposase-based tagging also enabled the development of ATAC-seq, which is a powerful technique for assessing chromatin accessibility at the genome level [23,128]. Additionally, combination of high-throughput sequencing with 3C-based approaches, including 3C, 4C, and Hi-C can reveal the three-dimension organization of viral chromatin and its interactions with the host genome, providing a spatial context for epigenetic remodeling [12,113]. Moreover, while traditional imaging techniques had limited sensitivity in detecting viral DNA, HSV-1 genomes pre-labelled with EdC enables the single-molecule detection of viral DNA by click chemistry, which revealed the positions of viral genomes relative to different histones with high resolution [13]. There are also techniques that have not been widely used in the HSV chromatin field yet but are likely to be useful for advancing the field in future. For instance, genome-scale CRISPR knockout or activation libraries can be employed to systematically uncover key epigenetic factors that control HSV chromatin. In addition, recently developed proteomic techniques such as proximity labeling [129] should allow the capture of potential key proteins that interact with chromatin particularly for weak or transient interactions that are often missed by traditional immunoprecipitation. Furthermore, while traditional methods mainly rely on analysis of bulk samples, recently developed single-cell multi-omics approaches have the potential of analyzing both gene expression and chromatin accessibility in individual cells and provide critical insights into cell-to-cell heterogeneity.

Development of infection models also contributes greatly to the evolving field, especially for the studies of latency and reactivation, which require non-proliferating neurons. Early studies of HSV chromatin during latency and reactivation relied almost exclusively on animal models (e.g., [31]). Although these models remain the gold standards for investigating latency mechanisms in the in vivo contexts, they are not easy to manipulate for procedures such as gene knockdown or drug treatment. Primary neuronal culture models that were developed later help solve this problem and have proven useful for understanding the regulatory mechanisms during these processes (e.g., [52]). Still, such models that use mouse or rat neurons may not faithfully recapitulate the interactions between the virus and its natural hosts, humans. To solve this issue, human neuronal models have been developed [130]. Notably, induced pluripotent stem cell (iPSC)-derived human neurons have recently emerged as an attractive model [131] because the differentiated neurons exhibit morphology and gene expression patterns comparable to human sensory neurons and support HSV-1 latency and reactivation. These cultures are also easy to propagate for applications requiring large numbers of cells. Nevertheless, they are artificially developed and may lack the full complexity of the in vivo environment. Therefore, the different models are complementary in their advantages, and results from all these models should be considered when we draw conclusions about particular mechanisms.

Despite the progress in this field, many questions remain. Also, new questions keep emerging as new techniques are being used. For example, while sophisticated imaging techniques reveal population heterogeneity in the spatial proximity of different histones at viral DNA, they raise questions as to the mechanisms and impacts of such heterogeneity [13]. Overall, the remaining questions in this field point to at least the following major directions for future research. (1) Mechanisms of viral chromatin assembly and maintenance during lytic infection. One knowledge gap in this direction pertains to host factors. Although iPOND has identified host factors associated with replicating viral DNA [17,132], the factors involved in earlier processes of chromatin assembly have not been systematically investigated. Also, to our knowledge, no genetic screen focusing on host epigenetic regulators has been conducted for HSV research. Systematic identification of host factors involved in viral chromatin assembly and maintenance may be needed before mechanistic exploration. (2) Mechanisms of chromatin regulation of HSV latency and reactivation. Given the neuron-specific nature of HSV latency, previous research in this direction has been constrained by the availability of convenient models, resulting in findings that were often descriptive with limited mechanistic insights. Recently developed models may help solve this problem. For example, iPSC-derived human neurons may be used for proteomics studies aimed at identifying host factors incorporated into viral chromatin during latency [131]. (3) Mechanisms of differential regulation of lytic and latent genes. Although recent studies involving CTCF have provided some insights into this issue, it remains incompletely solved. Besides ongoing work on insulators, future research could employ genome-wide sequencing approaches to map the occupancy patterns of various histone modifications across the viral genome and then focus on modifications exhibiting differential occupancies between lytic and latent genes as well as their upstream regulators. Exploring the three-dimensional structures of viral chromatin by 3C-based approaches may also help address this question.

Previous studies have made it clear that viral chromatin plays a critical role in multiple stages of the HSV life cycle. Importantly, it appears to be a major mechanism that enables HSV latency and controls the lytic–latent balance. With the availability of more sophisticated techniques, we are increasingly capable of delving deep into the mechanistic details. Many inhibitors targeting epigenetic processes have been developed and more are expected to emerge in future. Hopefully, the mechanisms revealed by future studies will pave the way for developing new therapeutics that can not only treat but also cure HSV-related diseases.

## Data Availability

No new data were created or analyzed in this study. Data sharing is not applicable to this article.
